# Pediatric Perioperative Stress Responses and Anesthesia

**Published:** 2017

**Authors:** Koichi Yuki, Erika Matsunami, Kazumasa Tazawa, Wei Wang, James A. DiNardo, Sophia Koutsogiannaki

**Affiliations:** 1 Department of Anesthesiology, Perioperative and Pain Medicine, Cardiac Anesthesia Division, Boston Children’s Hospital, Boston, USA; 2 Harvard Medical School, Boston, USA

**Keywords:** Cortisol, catecholamine, inflammation, anesthesia, immunological signature

## Abstract

Surgical stress responses cause an array of endocrinological, metabolic and immunological changes in patients. The landmark studies in the 1980s showed that adequate anesthesia dramatically improved the outcomes of pediatric surgical patients by attenuating stress hormonal responses, pointing out the harm of ‘inadequate’ anesthesia. Subsequent studies questioned the role of administering very high-dose anesthetics to further attenuate stress responses. Here we review the feature of surgical stress responses in pediatric patients including their difference from those in adult patients. Overall, pediatric patients show minimal or no resting energy expenditure change postoperatively. In adult patients, increased resting energy expenditure has been described. Pediatric patients demonstrated robust cortisol and catecholamine responses than adult patients. However, the duration of these surges is often short-lived. Systemic proinflammatory and anti-inflammatory cytokine levels have been measured. Pediatric patients showed less proinflammatory cytokine elevation, but had similar anti-antiinflamatory responses. We also review in detail the immunological changes in response to surgical stress. Based on our current knowledge, we attempted to understand the underlying mechanism how adequate anesthesia dramatically improved the outcome of patients. Although more work is needed to be done, understanding how pediatric patients respond to perioperative stress, and its mechanism and consequence will allow us to direct us into a better, perioperative management in this population.

## Introduction

Stress is defined as stimuli that cause disequilibrium to an organism and therefore threaten homeostasis [1, 2]. When the human body faces stress such as an injury or a trauma, both the hypothalamic-pituitary-adrenal axis (HPA) and the sympathetic nervous system become activated and a range of metabolic, endocrinological and immunological responses occur [3]. Presumably these responses are developed for survival benefit. By extrapolation, surgery is considered to be a “trauma” and induces stress responses. Our intent here is to review surgical stress responses in pediatric patients. Because the physiology of newborns, children and adults differs at their baseline due to their developmental differences, subtle differences in stress responses are likely to exist between pediatric and adult patients. We also review the role of modifying surgical stress response for patient outcomes.

## Perioperative stress responses

**Figure 1:**
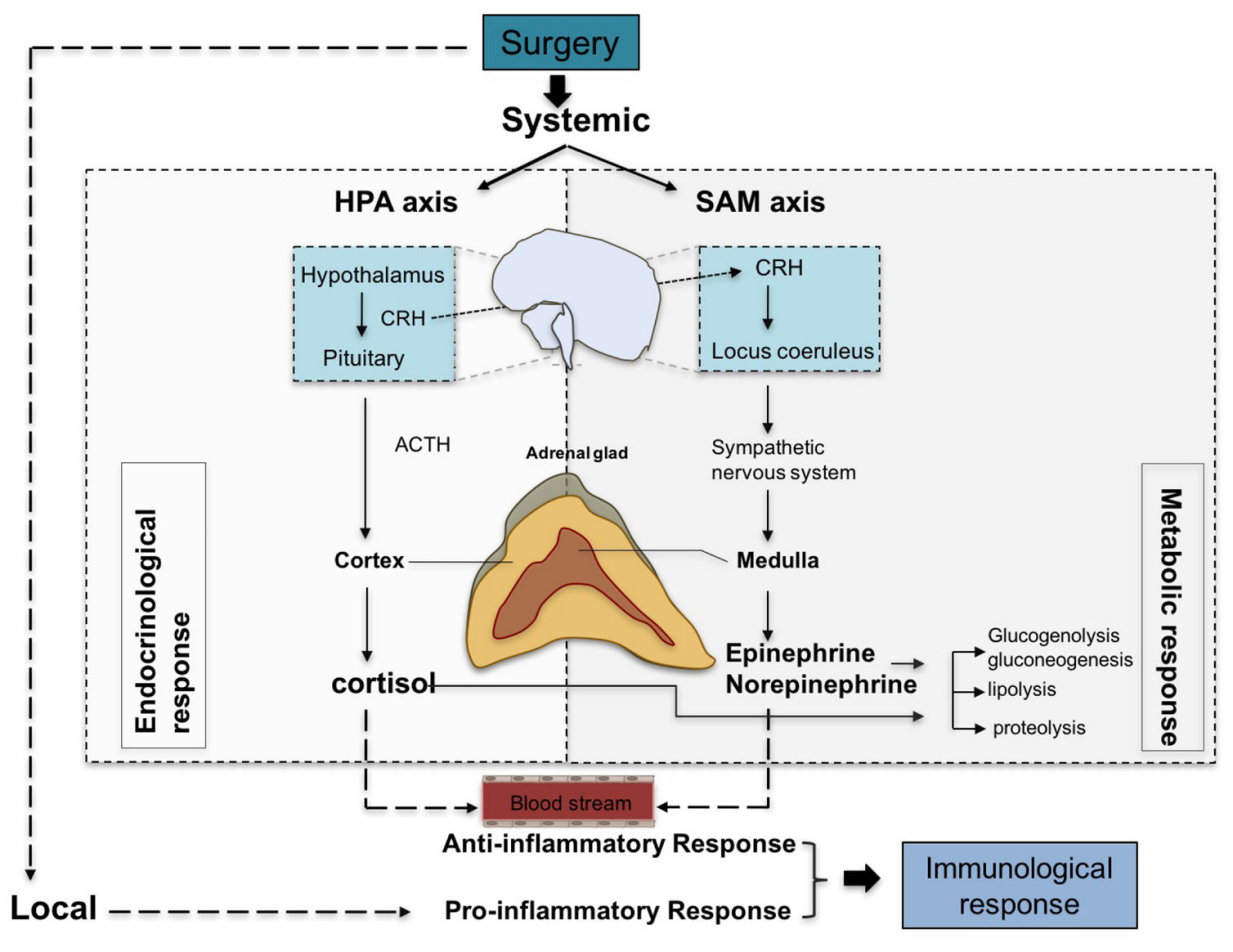
Perioperative stress responses Upon stress, such as surgery, both local and systemic responses are triggered leading to pro-inflammatory and anti-inflammatory events, respectively. Systemic response involve the stimulation of HPA and SAM axis and result in a cascade of endocrinological and metabolic responses through the production of cortisol and catecholamines (epinephrine, norepinephrine). These “stress hormones” can activate immune cells in the blood stream leading to the production of anti-inflammatory cytokines. Local immunological responses on the other hand accompany inflammatory responses including pro-inflammatory cytokine production. CRH; corticotropin-releasing hormone, ACTH; adrenocorticotropic hormone.

The presumed role of the stress responses is to prevent secondary damage and increase the availability of substrates required by essential organs and healing tissues. Not only surgical stimulus, but also temperature change, blood loss and altered blood flow pattern can trigger the stress response. A good example is surgery performed with the assistance of cardiolpulmonary bypass (CPB), where hypothermia, contact activation, hemodilution, and nonpulsatile flow are involved. Not surprisingly, cardiac surgery is a major stimulus of massive stress responses. Stress responses lead to metabolic, endocrinological, and immunological changes. A surgical insult triggers a central response via afferent nerves to activate both the HPA axis and the sympathetic-adrenal-medullary (SAM) axis. In addition, it triggers a local response including cytokine production. Cytokines produced locally can act on the central nervous system. These complex processes are illustrated in Figure 1.

Contrary to our presumption that the stress responses had evolved to promote survival, earlier studies in pediatric patients demonstrated that attenuation of surgical stress responses was associated with improved outcomes, reduced complications, and faster recovery time [4–9]. Some speculated that our stress response had not been fine-tuned to mitigate severe trauma and surgical stress [1]. Excessive stress responses can lead to systemic inflammatory response (SIRS) and prolonged catabolism of body stores [10]. In contrast, an extended period of postoperative immunoparalysis can predispose to secondary infection. Ideally excessive responses to surgical stress would be mitigated to prevent SIRS while at the same time allowing those inflammatory responses responsible for the initiation of reparative healing to occur. We will review the underlying mechanism of these opposing responses with surgical stress.

## The characteristics of surgical stress responses in pediatric patients

### Endocrinological and metabolic stress responses

The main feature of endocrinological stress response is a release of “stress hormones” such as catecholamines, cortisol and glucagon via the activation of the HPA axis and the SMA axis. They trigger a cascade of metabolic responses to break down protein, fat and carbohydrate and mobilize resultant substrates for energy sources. Cortisol, catecholamines and glucagon shift from the production of structural proteins to acute phase proteins, and facilitate mobilization of stored glycogen, gluconeogenesis and lipolysis. Protein catabolism is stimulated by cortisol. Cortisol and catecholamines stimulate glycogenolysis and gluconeogenesis. Lipolysis is facilitated mainly by catecholamines, which convert triglyceride to glycerol and fatty acids. Glycerol is used for gluconeogenesis and fatty acids are for ketogenesis. Metabolites originating from fat are main energy sources in the perioperative period [11].

The activation of the HPA axis and the SMA axis can be assessed by measuring plasma stress hormone levels without difficulty. In contrast, an individual evaluation of protein, lipid and glycogen metabolism often requires more resources and such studies are limited so far. Metabolic responses have often been assessed with the use of indirect calorimety to obtain resting energy expenditure (REE) instead. Thus the degree of surgical stress responses has been gauged by measuring serum catecholamine and cortisol levels in many studies. Chernow et al. reported that stress hormonal responses correlated with the severity of surgical stress in adult patients [12]. In the ‘minimal’ surgical stress group (such as inguinal hernia repair), they were negligible. In the ‘moderate’ (such as cholecystectomy) and ‘severe’ (such as subtotal colectomy) surgical stress groups, plasma cortisol and norepinephrine levels were elevated at postoperative 1 and 24 hours, and epinephrine level was elevated at postoperative 1 hour. Similarly in adult patients who underwent cardiac surgery, cortisol level remained elevated at 24 hours after surgery [13–15]. In contrast, the salient feature of pediatric, particular neonatal stress hormonal responses is that the elevation of these stress hormones lasts for a shorter duration with a greater magnitude. Anand et al. studied neonates undergoing surgery (type of surgery was not specified in the study) and noted that plasma epinephrine and norepinephrine concentrations were significantly elevated at the end of surgery, but returned to their preoperative levels by 6 hour after surgery [16]. The same group studied a group of neonates undergoing surgery with different severity and measured several metabolic and endocrinological markers preoperatively and during the first 24 hours postoperatively [17]. Plasma epinephrine and norepinephrine concentrations were significantly elevated at the end of surgery. Epinephrine level was also elevated at 6 hours after surgery and then returned back to its baseline level. The hormonal responses in neonates were proportional to the degree of surgical stress. Cardiac surgery, particularly the one involving hypothermic CPB or deep hypothermic circulatory arrest (DHCA) was associated with greater and more prolonged stress hormonal changes, which is in line with the study in adult patients by Chernow et al [17]. The peak epinephrine and norepinephrine levels in neonates undergoing cardiac surgery were 1.4 ~ 7.2 and 3.4 ~ 10.2 times higher than those in adult patients, respectively [18, 19]. The study by Boix-Ocha et al. demonstrated that plasma cortisol level went up significantly (up to 1000 nmol/L) in neonates and infants intraoperatively, and returned back to its baseline value in the very early postoperative period [20]. Interestingly, the pattern of these stress hormonal responses was prevalent and characteristic in pediatric patients at various ages [21]. Overall, in the pediatric population, catecholamine and cortisol levels tended to be much higher intraoperatively, but returned to their baseline levels by the very early postoperative period, while they remained elevated for a longer duration postoperatively in the adult population [22].

Studies associating these cortisol and catecholamine responses with lipid, protein and carbohydrate metabolism separately have been limited. It was recent advanced techniques using nuclear magnetic resonance, spectroscopy, and mass spectrometry have been applied to metabolic profiling [23]. Thus many studies measured REE by indirect calorimetry in conjunction with the measurement of plasma stress hormones. Studies measuring REE by indirect calorimetry suggested that increase in the REE in pediatric patients was not sustained for prolonged periods as in adult patients. Postoperative REE changes in adult patients were well studied by Cuthbertson and others, and were characterized as an initial reduction in metabolic rate (‘ebb’ phase) associated with reduced cardiac output and lactic acidosis (the first 24–48 hours postoperatively) followed by an increase (‘flow’ phase) up to several days after surgery [24]. In contrast, the majority of pediatric studies demonstrated little or no postoperative REE increase with any increase lasting only for a very brief period (< 12–24 hours postoperatively) [25–29]. Based on these findings, it was postulated that infants and children would divert protein and energy from growth to wound healing without an increase in energy expenditure [27]. Metabolic profiling using proton nuclear magnetic resonance spectroscopy demonstrated that ketone bodies (acetone, acetoacetate and 3-D-hydroxy-butyrate) were elevated postoperatively [23]. This was likely a consequence of cortisol and catecholamine induced lipolysis and ketone bodies production. Further detailed assessment of the relationship between hormonal changes and lipid, protein, and carbohydrate profile dynamics in the perioperative setting will enhance our understanding of surgical stress responses.

## Immunological responses

A surgical insult initiates a series of immunological responses. These can be largely divided into the proinflammatory responses aimed at eradicating any causal agents and secondary opportunistic microbial invasion, and systemic deactivation of the immune system to restore homeostasis, which occasionally progresses into the extreme called ‘immunoparalysis’. Overall, adequate immunological responses protect against infection, provide effective wound healing, and are key determinant of postoperative recovery.

**Figure 2:**
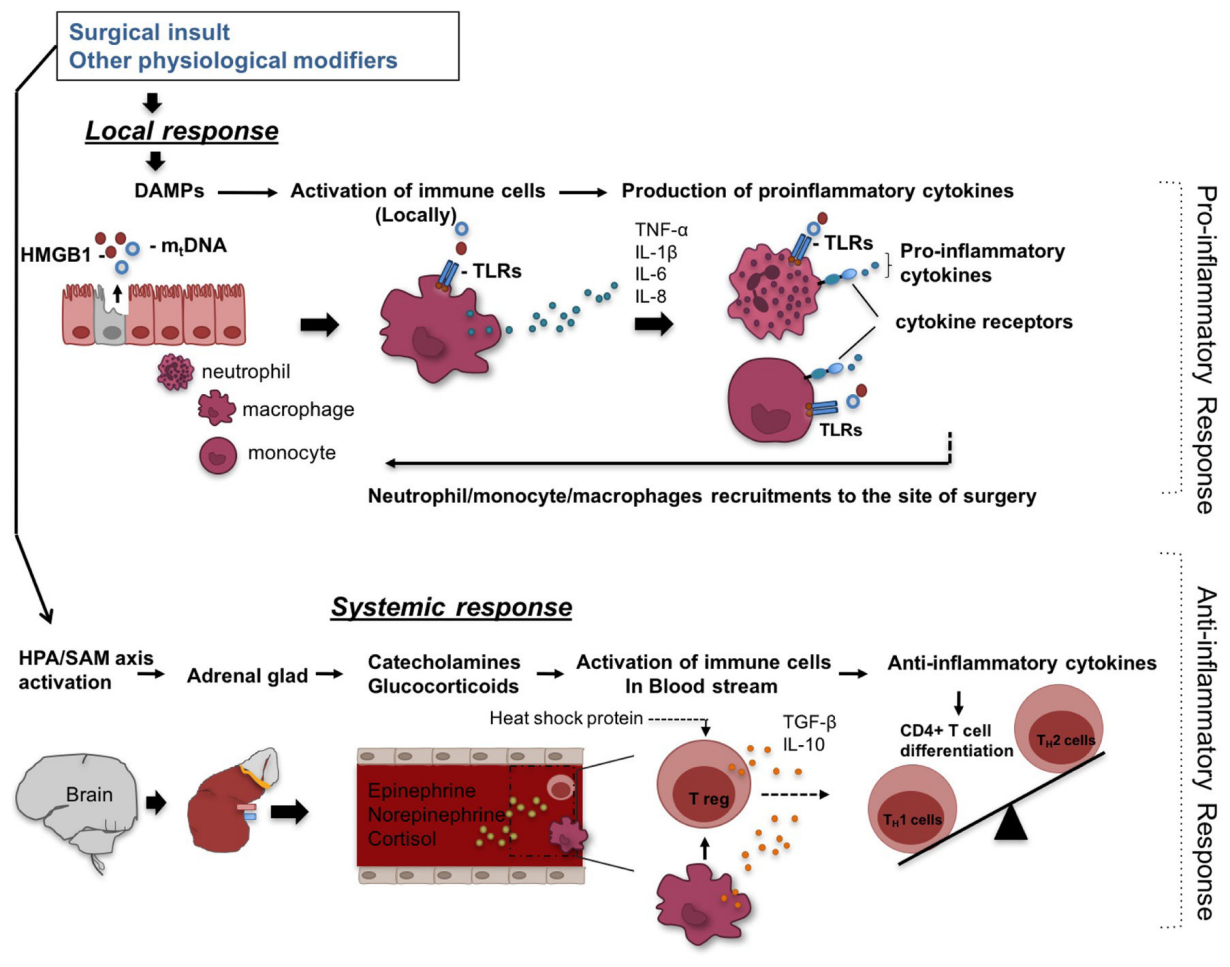
Surgery-induced Immunological responses Surgery can induce local response manifested by release of damage- associated molecular patterns (DAMPs) such as high mobility group box 1 (HMGB1) and mitochondrial DNA (mtDNA), which through toll-like receptors (TLRs) stimulate macrophages/monocytes to produce pro-inflammatory cytokines such as tumor necrosis factor (TNF)-α, interleukin (IL)-1β, IL-6 and IL-8. In turn, these cytokines together with DAMPs activate and recruit neutrophils and macrophages/monocytes to inflammatory sites. In addition to this local response, surgery can triger systemic responses through stimulation of the hypothalamic-pituitary-adrenal (HPA)/sympathetic-adrenal- medullary (SAM) axis and subsequent production of cortisol and catecholamines (epinephrine and norepinephrine). These hormones activate immune cells in the blood stream to produce anti-inflammatory cytokines. Anti-inflammatory cytokines such as transforming growth factor (TGF)-β and IL-10 induce regulatory T cells (Treg) cells, favoring type 2 T helper (Th2) responses (anti-inflammatory). Heat shock proteins are also produced during surgery-induced stress amplifying Treg function.

The immunological alternation in the perioperative setting derives from a combined result of local and central events (Figure 2). With surgical insult, host molecules called damage-associated molecular pattern molecules (DAMPs) or alarmins are released from necrotic cells and induce inflammation. Pathophysiological changes such as ischemia-reperfusion injury also contribute to the release of DAMPs [30]. DAMPs include high-mobility group box 1 (HMGB1) and mitochondria DNA, and stimulate innate immune cells such as macrophages/monocytes to produce proinflammatory cytokines. HMGB1, a nuclear protein that modulates transcription, is also categorized as a cytokine and secreted by activated macrophages and other immune cells[31]. High-mobility group box 1 protein (HMGB1. Mitochodria DNA is released from damaged cells and is detectable in the blood stream following a significant injury [32]. Toll-like receptors (TLRs) are pattern-recognition receptors that recognize both infectious materials and DAMPs, and are major receptors to induce the production of proinflammatory cytokines in the face of DAMPs [33–35]. Proinflammatory cytokines such as tumor necrosis factor (TNF)-a, interleukin (IL)-1b, IL-6 and IL-8 are primarily secreted from monocytes and macrophages. Together with DAMPs, they activate and recruit neutrophils and monocytes to inflammatory sites by interacting with cytokine receptors and TLRs [36, 37]. In addition to this local response, surgical insult stimulates the HPA axis and the SAM axis via the afferent nerves to lead to the systemic secretion of cortisol and catecholamines. Glucocorticoid receptors are expressed in neutrophils, monocytes, macrophages, T cells and B cells and cortisol shifts them to the cells with anti-inflammatory phenotype [38]. Catecholamine receptors are found in monocytes, macrophages, natural killer (NK) cells, B cells and T cells, and their stimulation induces anti-inflammatory responses [39]. Anti-inflammatory responses are induced most potently by epinephrine, followed by norepinephrine, and least by cortisol [40]. Anti-inflammatory cytokines such as IL-10 and transforming growth factor (TGF)-b induce regulatory T cells, a subset of Cluster of differentiation (CD) 4^+^ T cells with suppressive activity, from a pool of CD4^+^ T cells [41], and these regulatory T cells also bias CD4^+^ T cells toward Th2 cells, which are anti-inflammatory [42] (Figure 2). In addition, heat shock proteins (HSPs), chaperone proteins released under stress, amplify regulatory T cell function [43, 44]. Thus, surgically injured tissues demonstrate proinflammatory responses, while leukocytes in blood stream are anti-inflammatory and hyporeactive [45]. Adaptation to surgical stress involves coordinating local inflammation with systemic anti-inflammation so as to allow concentration of activated phagocytes and other effectors only at injured local site [46]. In some pathophysiological conditions such as contact activation due to CPB use and ischemia-reperfusion injury, DAMPs can be present in the systemic circulation and also induce systemic proinflammatory responses (Figure 2).

The perioperative leukocyte distribution was studied in detail in adult patients undergoing surgery [47]. Postoperative increase in neutrophil counts peaked at postoperative day 1 with no difference in monocyte and B cell counts, and reduction in various types of T cell counts peaking at 12 hours after surgery was seen. Regulatory T cell population, induced by anti-inflammatory cytokines, expanded at one week after surgery [48]. Some of other studies found monocyte counts to decrease or increase postoperatively [49, 50]. Postoperative lymphopenia was also reported in the pediatric population with its peak at 12 hours after surgery, as in the case in adults [21, 51]. The distribution of immune cell subsets in the perioperative period seems to be regulated at least in part by stress hormones [52]. Epinephrine and norepinephrine induce a redistribution of immune cells from spleen, bone marrow and the marginated pool into the bloodstream and temporarily increase blood leukocyte counts [53]. Subsequently cortisol and epinephrine induce the movement of immune cells out of the blood stream to surgical site or back to their origins. How stress hormones control leukocyte tissue/blood distribution is not described yet. Because the pattern of catecholamine and cortisol elevation in pediatric population is different, there may be a subtle difference in the time-course of blood leukocyte counts between pediatric and adult patients.

The majority of perioperative, immunological studies have focused on measuring the level of serum proinflammatory and anti-inflammatory cytokines. It is intuitively obvious that systemic responses can differ depending on the type and duration of surgery, co-morbidities and additional modifiers such as ischemia-reperfusion and CPB use. SIRS is one of the maladaptive arms of stress responses and involves excessive systemic inflammation. Contact activation by CPB and ischemia- reperfusion injury, for example, can produce systemic DAMPs, activate monocytes, and stimulate systemic release of proinflammatory cytokines, resulting in SIRS. Systemic inflammation can cause endothelial activation and subsequent endothelial-leukocyte interaction. Plasma TNF-a and IL-1b levels increase early. This is followed by IL-6 and IL-8 elevation. Plasma IL-1b is often elevated in adult cardiac surgical patients [54, 55]. However, IL-1b was not necessarily detected after major surgery including cardiac surgery in children [56, 57]. In adult patients, plasma TNF-a was not detected in minor surgery, but was elevated in major surgery [58]. Uncomplicated major surgery in pediatric patients was not associated with elevated levels of TNF-a [59]. TNF-a facilitates leukocyte-endothelial interaction, and its elevation in infants postoperatively was correlated with capillary leak syndrome [60]. IL-6 is the most consistently elevated cytokine in blood in the postoperative period [1]. A change in plasma IL-6 level became significant after 2 to 4 hours following surgery, peaking at 6 – 24 hours [3]. Overall systemic proinflammatory response seems to be attenuated in small children [61]. This might be explained partly by more robust elevation of catecholamines and cortisol in the pediatric patients. In addition, the developmental change of TLR-mediated responses should be considered. TLR-mediated responses are age-dependent; anti-inflammatory responses are more dominant at the age < one year, and proinflammatory responses become dominant over time [34]. As opposed to the systemic proinflammatory responses, the systemic anti-inflammatory responses do not seem to differ between children and adults. Early responders among anti-inflammatory cytokines include IL-10, soluble IL-1 receptor antagonist (IL-1Ra), TNF soluble receptors 1 and 2 (TNFsr1 and 2), and TGF-b [55]. In adult cardiac surgical patients, IL-10 and IL-1ra peaked soon after the termination of CPB, followed by an increase in TNFsr1 and 2. IL-10 and IL-1ra returned to their baseline levels at 24 hours after surgery [62, 63]. Similar responses have been observed in pediatric patients undergoing cardiac surgery [60, 64].

The phenotype of monocytes has been studied, particularly their human leukocyte antigen (HLA)-DR surface expression. HLA-DR is a component of major histocompatibility complex (MHC) class II and is involved in antigen presentation to T cells. IL-10 induces an accumulation of MHC class II complexes in intracellular vesicles and reduces their surface expression [65]. Reduction of HLA-DR surface expression can occur in response to anti-inflammatory cytokine milieu under a surge of stress hormones. Postoperative reduction of HLA-DR surface expression on monocytes has been reported in adult and pediatric patients [62, 66]. However, baseline profile of their HLA-DR surface expression may not be the same between pediatric and adult patients. Kanakoudi-Tsakalidou et al. reported that healthy neonates had significantly lower HLA-DR positive monocytes than adult (69% versus 91.5%) [67], suggesting that HLA-DR expression might be influenced by other factors as well.

## Surgical stress responses and anesthesia

### Historical perspective of anesthesia and stress responses in pediatric patients

For decades, it was believed that neonates could not feel pain, and they often underwent surgery without adequate anesthesia on our current standard. As recently as the1980s, it was reported that 77% of newborn babies undergoing surgical ligation of patent ductus arteriosus (PDA) received either muscle relaxants alone or with nitrous oxide [16]. The landmark studies by Anand et al. in the mid 1980s led to reconsideration of anesthetic management for neonatal surgery [4]. The outcomes of 16 preterm babies undergoing PDA ligation under nitrous oxide with or without fentanyl (10 μg/kg) were compared. They demonstrated that intraoperative use of fentanyl significantly improved perioperative outcomes (less postoperative ventilator support, less hemodynamic and metabolic complications) consistent with the concept that neonates could feel pain. Plasma epinephrine, norepinephrine and cortisol levels were significantly elevated up to 24 hours after surgery in neonates who did not receive fentanyl. Blunting hormonal response was presumably responsible for this improved outcome. Anand et al. also examined 36 neonates (27 term, 9 preterm) undergoing non-cardiac surgeries under nitrous oxide with or without halothane (1–2% of halothane for induction, 0.5–1% for maintenance) [6]. The peak epinephrine, norepinephrine and cortisol levels were significantly lower in the halothane group. The group who did not receive halothane had higher postoperative complications including gastric bleeding, arrhythmias, poor peripheral perfusion, increased ventilatory support, oliguria, and paralytic ileus. The importance of anesthesia was further examined in cardiac surgical patients. Eliis and Steward reviewed the charts of 36 patients (average 4.7 year old) who had undergone cardiac surgery with hypothermic CPB or profound DHCA under fentanyl anesthesia (7 to 88 μg/kg) [68]. They found that elevated blood glucose was associated with poorer neurological outcome following DHCA, and fentanyl attenuated the hyperglycemia associated with hypothermic CPB and DHCA. Anand and Hickey compared neonates who received deeper anesthesia consisting of high doses of sufentanil (37 μg/kg) and postoperative infusions of fentanyl or sufentanil for 24 hours with neonates who received lighter anesthesia with halothane (0.5%) and morphine as needed [5]. The group with deeper anesthesia had attenuated hormonal stress responses with fewer incidences of sepsis, metabolic acidosis, disseminated intravascular coagulation, and postoperative deaths. These studies established the concept that provision of adequate anesthesia to pediatric patients is critical to attenuate stress response for better outcomes. The endocrine control of metabolic homeostasis is believed to be already functional at 16 weeks’ gestation [69, 70].

### Outcome assessments following surgical stress and their potential markers

Attenuating surgical stress responses to improve perioperative outcomes of pediatric patients by providing adequate anesthesia represented a significant paradigm shift in our clinical practice. Compared to the “common” practice two-three decades ago, it is without doubt that our perioperative anesthetic management of pediatric patients has improved significantly in conjunction with the improvement of monitoring, surgical technique and postoperative care, and a better understanding of disease pathophysiology. Now a new question is - how much should we attenuate stress responses? Duncan et al. evaluated the effect of different doses of fentanyl (2, 25, 50, 100, or 150 μg/kg) in 40 infant and children (0.3 to 44 months) undergoing elective cardiac surgery [71]. Patients receiving the lowest fentanyl dose experienced a significant elevation in glucose, cortisol and norepinephrine levels. Anesthetic regimen containing fentanyl 25–50 μg/kg was sufficient to blunt hemodynamic and hormonal stress responses. Higher doses of fentanyl (100 and 150 μg/kg) offered little advantage over more a moderate dose of fentanyl (50 μg/kg) in stress hormonal responses and were associated with hemodynamic compromise. There was no significant difference among the five groups in regard to time to extubation, the duration of intensive care unit (ICU) stay, and the incidence of infection or other postoperative complications. Gruber et al. examined 45 children (average 3 months) undergoing elective cardiac surgery under three different anesthetic regimens [72]. The first group received fentanyl boluses of 25 μg/kg at four time points during the surgery, the second group received fentanyl 25 μg/kg bolus followed by 10 μg/kg/h infusion, and the last group received fentanyl infusion at 10 μg/kg/h and midazolam at 100 μg/kg/h. Cortisol, epinephrine, and norepinephrine levels were best attenuated in the group that received the highest dose of fentanyl. Also there was no significant difference among the three groups in the duration of mechanical ventilation, ICU or hospital stay or postoperative complications. These studies questioned the benefit of overly attenuating stress responses by using high dose of opioids.

The underlying mechanism whereby adequate anesthesia, defined as attenuation of the neurohormonal responses, improves patient outcomes, is not known and is ethically impossible to try to determine at this point. However, considering that cortisol and catecholamine levels in patients with what is now considered inadequate anesthesia were significantly elevated in the perioperative period, it is likely that these patients had exaggerated anti-inflammatory responses with resultant immunoparalysis. And immunoparalysis could lead to increased complications. Attenuation of the neurohormonal stress responses likely resulted in better balance of proinflammatory and anti-inflammatory responses. However, complete abolition of hormonal stress responses will not be beneficial either as inadequate stress hormonal responses can be detrimental in patients with adrenal insufficiency.

**Figure 3:**
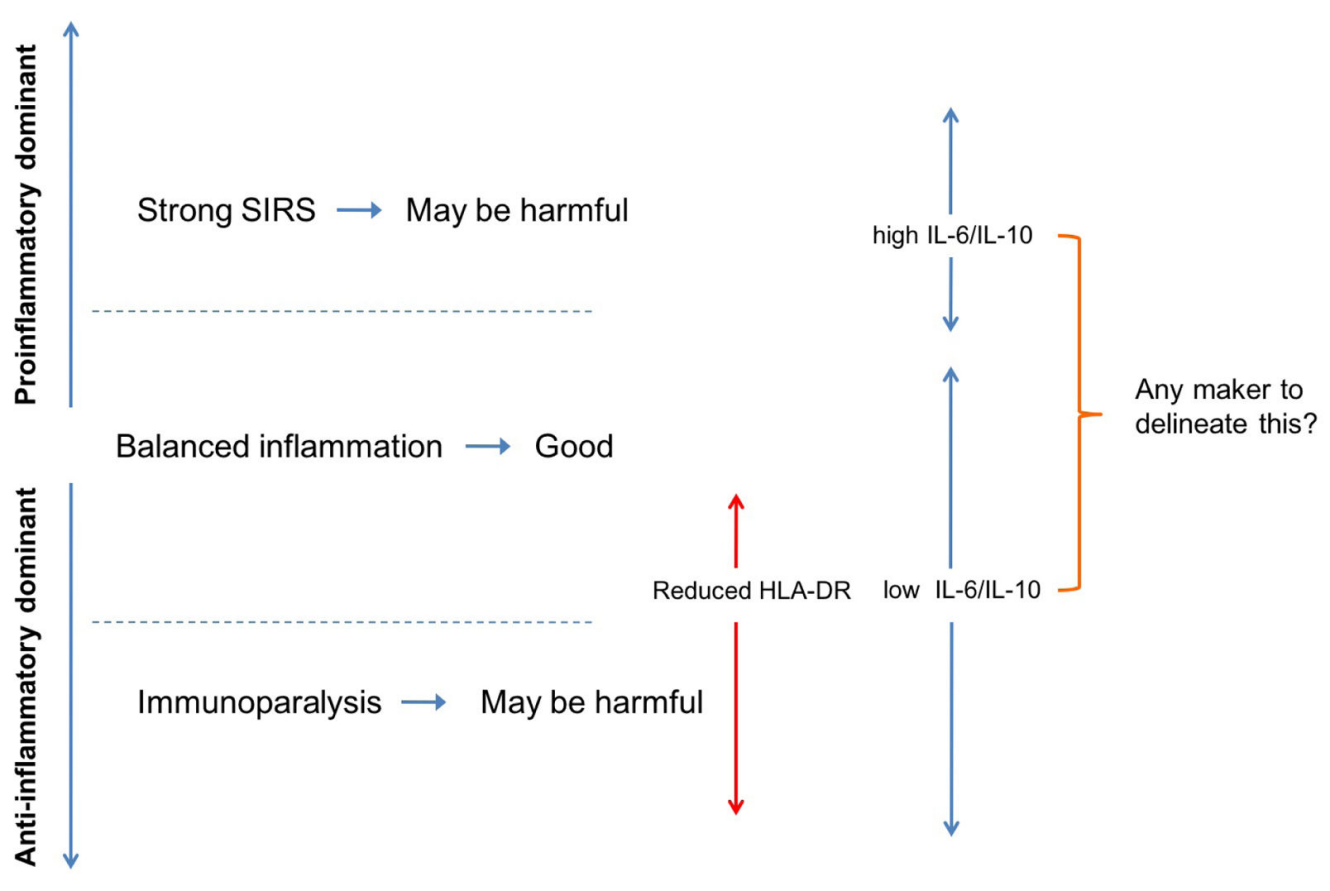
Balance between proinflammatory and anti-inflammatory responses Both excessive systemic inflammatory response syndrome (SIRS) and excessive immunodepression (immunoparalysis) may be harmful to patients. The relationship with IL-6/IL-10 ratio and human leukocyte antigen (HLA)-DR expression with proinflammatory/anti-inflammatory balance is shown.

While assessment of stress hormonal responses to surgical stress has been a common method to assess the degree of the stress response, it does not provide the complete, phenotypic picture. Consequently changes in other parameters induced by surgical stress have been explored. The immunological signature is complex and the recent data suggests that the immunological signature induced by surgical stress can predict recovery [50]. Two signatures are well studied. One is the ratio of IL-6/IL-10 and the other is HLA-DR surface expression on monocytes. The IL-6/IL-10 ratio is considered as a surrogate of the balance between systemic inflammation and anti-inflammation. In patients with sepsis, higher IL-6 to IL-10 ratio was correlated with poor outcomes [73]. Conversely, a reduction in IL-6 to IL-10 ratio in infants undergoing cardiac surgery was predictive of better outcomes [74]. Although anti-inflammatory cytokines limit the extent of inflammatory responses and aid in restoration of homeostasis, excessive systemic anti-inflammatory responses, which reduce IL-6 to IL-10 ratio, may cause immunoparalysis and poorer outcomes [75]. HLA-DR surface expression on monocytes has been also studied in relationship to patient outcomes because the reduction in HLA-DR surface expression on monocytes has been considered to be a marker of immunodepression [76–79]. In adult cardiac surgical patients, the reduction in HLA-DR expression was associated with poorer clinical outcomes [62]. Similarly, HLA-DR expression was reduced postoperatively in pediatric cardiac surgical patients and HLA-DR expression of < 60% of monocytes was associated with an increased risk factor of SIRS/sepsis and prolonged ICU stay [66]. In addition, low HLA-DR expression within the first 72 hours was an independent predictor of postoperative sepsis. Among monocytes with reduced HLA-DR expression, changes in signal transducer and activator of transcription 3 (STAT3), NF-kB and adenosine 3′,5′-monophosphate response element-binding protein (CREB) were strongly associated with postoperative recovery profile of patients who underwent hip arthroplasty [50]. *Ex vivo* TLR4 signaling in monocytes were tested in the same population and replicated these signature changes with postoperative recovery [80]. STAT3 is a very interesting molecule that regulates the production of both proinflammatory cytokine IL-6 and anti-inflammatory cytokine IL-10 along with TLR [81], and may be worth studying as a potential marker. While HLA-DR expression on monocytes is an appealing marker to predict perioperative outcomes, it may be a very sensitive, but not specific marker as reduced HLA-DR expression has been observed on monocytes from patients who underwent low-intermediate risk surgery [82], Optimistically makers to detect balanced immunological profiles may allow us to direct our perioperative stress response approach (Figure 3). To understand how different anesthetic agents and techniques modulate surgical stress responses is needed to improve our perioperative management and outcome. While some anesthetics may alter hormonal stress responses by altering the central nervous system, other anesthetics not only affect hormonal responses, but also affect immune cells and can change the immunological signature of immune cells directly [83–85]. In addition to general anesthesia, regional anesthesia potently suppresses surgical stress responses by afferent and efferent sympathetic blockade [86]. An optimal immunological marker, if available, can be used to assist our perioperative anesthetic management.

In summary, the pediatric surgical stress response profile is different from that in adults; little of less postoperative REE postoperatively, robust cortisol and catecholamine surges with shorted duration, and less systemic proinflammatory cytokine responses. And less proinflammatory cytokine responses may be explained by robust cortisol and catecholamine responses. Current anesthesia regimens have improved outcomes of pediatric patients by suppressing extreme stress responses. Future research is necessary to understand both how to optimize modulation of stress responses and to find accurate markers for optimal modulation.
